# Solar-driven water-splitting provides a solution to the energy problem underpinning climate change

**DOI:** 10.1042/BST20200758

**Published:** 2020-11-26

**Authors:** James Barber

**Affiliations:** Department of Life Sciences, Sir Ernst Chain Building-Wolfson Laboratories, South Kensington Campus, Imperial College London, London SW7 2AZ, U.K.

**Keywords:** energy cycle, hydrogen, photosynthesis, photosystem II, water-splitting

## Abstract

The emergence of the oxygen-evolving photosystem two complex over 2.6 billion years ago represented the ‘big bang of evolution’ on planet Earth. It allowed phototrophic organisms to use sun light as an energy source to extract electrons and protons from water, and concomitantly release oxygen. Oxygenic photosynthesis not only created an aerobic atmosphere but also removed CO_2_ to produce the organic molecules that make up the current global biomass and fossil fuel. In addition, it paved the way for animal life. Today extensive burning of fossil fuels is reversing the results of photosynthesis through billions of years, rapidly releasing CO_2_ back into the atmosphere and consequently increasing the temperature of the planet. There is an urgent need to develop new sustainable energy sources, but the choice is not obvious. My approach to this problem has been to unravel the blueprint of photosystem II (PSII) and to develop an ‘Artificial Leaf’ technology. A significant step with respect to that mission was achieved at Imperial College when we could conclude from X-ray diffraction of PSII crystals, that the water-splitting catalytic centre consists of a unique Mn_3_Ca^2+^O_4_ cubane structure with a fourth dangler Mn oxo-bonded to the cubane. Here I use this and more recent structures to discuss the mechanism of water splitting and O–O bond formation. Furthermore, I will address how this information can be used to design novel water-splitting catalysts and highlight recent progress in this direction. My conviction is ‘if plants can do it, we can do it — after all it is all about chemistry’.

## Introduction

I feel extremely honoured to have been awarded the Heatley Medal and Prize. I share this honour with those who have worked with me over the years to elucidate the molecular details of the photosynthetic process. Most of my research has focussed on photosystem II (PSII), a biological machine able to use light to split water into oxygen and reducing equivalents (protons and electrons). One can say that the emergence of PSII about 2.6 billion years ago was the Big Bang in Evolution; it created aerobic conditions on Planet Earth that paved the way for animal life, and converted CO_2_ into organic molecules that constitute our global biomass and fossil fuels. The process of photosynthesis relates directly to the challenges we today are facing with the sustainability of our Planet's energy supply and the serious issue of Climate Change. Our modern and industrialised societies are burning ever-increasing amounts of fossil fuels, thereby reversing the photosynthetic process through billions of years, and rapidly releasing CO_2_ back into the atmosphere and thereby increasing the temperature of the Planet.

Just about the time I started writing this paper, there seemed to be a surge of recognition by the general public in the UK that the emission of greenhouse gases into the atmosphere (particularly CO_2_) would lead to global warming and serious climate change and that the situation was urgent. It seemed that the trigger for this was a television documentary narrated by David Attenborough (‘Climate Change — The Facts’ first shown April 18, 2019) and a 15-year-old school girl from Sweden, Greta Thunberg, who, through social media and protesting outside the Swedish Parliament Building, urged school children to ‘strike’ weekly to demonstrate about the lack of action by policy makers to respond urgently to this threat. Prior to this, many high profile people had been warning the UK Government of the seriousness of this situation, including David King, UK Chief Scientific Advisor (2000–2007), and Robert May and Martin Rees, former Presidents of the Royal Society (2000–2005 and 2005–2010, respectively). Indeed, Martin Rees expressed his views in a recent book he published, ‘On the Future: Prospect for Humanity’ [1], while David King was involved in initiating the 2008 UK Climate Change Act when Ed Miliband was Secretary for State for Energy and Climate Change.

Despite the negativity of the present administration in the US to accept the urgency of action on climate change, there is a long history of concern and lobbying. There are many key individuals such as the ex-Vice President of the US, Al Gore, and academics like James Hansen (NASA and University of Columbia), Nobel Laureate William Nordhaus (Yale University), Nathan Lewis (CalTech), Steve Chu (ex-US DOE Secretary for Energy (Washington-Obama administration/Stanford and Nobel Laureate). In addition, there is the host of scientific authors of the many in-depth reports of the Intergovernmental Panel on Climate Change (IPCC) from 1988 when it was formed. Despite this, over the years, the US administrations have not had the courage to address the challenging problems associated with moving away from business as usual to avoid the predicted devastating consequences of global warming. For example, which government in the US would have the guts to put up the price of petrol or diesel to match the prices in Europe or even higher? And which administration would withstand the pressure from oil companies like Exxon to change the status quo?

The solution now is far more complicated with the IPCC insisting we move to a global target of zero emissions of CO_2_ by 2050 in order to prevent no further increase in atmospheric temperature beyond 1.5°C (that is 2.5°C above pre-industrial levels.).

## Global bioenergetics

It was the Swedish chemist and Nobel Laureate Svante Arrhenius who realised in about 1900 that the CO_2_ being released into the atmosphere at that time due to industrialisation would ultimately bring an increase the temperature of our Planet [2]. Probably because he lived in Sweden, he suggested that this global warming would be a benefit — not only making life more comfortable but because plants, especially crops, would prosper, so helping with the feeding of the ever-increasing world population. At that time, the atmospheric CO_2_ level was ∼280 ppm while today it is at a record level of 415 ppm as measured at the Mauna Loa Observatory in Hawaii in May 2019 [3]. A disturbing feature is that despite efforts to mitigate CO_2_ release into the atmosphere by increasing the use of power from wind, hydroelectric, nuclear and other renewables sources, the global CO_2_ atmospheric level continues to increase by ∼0.5% a year. This continuous increase in CO_2_ levels reflects the growth in population which is at present about 1.3 billion every 13 years [4], and in energy consumption per capita which increases with an improvement in living standards measured by the human development index (HDI). At present, the rate of energy usage globally corresponds to ∼18 TW averaged over a year [5]. Since the world population is 7.6 billion then every human being (man, woman and child) is continuously powered by 2.37 kW, equivalent to ∼24 × 100 W incandescent electric light bulbs. Of course, the distribution is not even, with an average European citizen powered by at least 10 times this corresponding to 250 × 100 W electric light bulbs, while the energy consumption per capita in the US is somewhere in the region of three times higher. The amount of power used to drive the metabolism of one human is ∼100 to 200 W.

When the Paris Agreement was adopted and ratified by 185 countries, the IPCC wrote a special report on how humanity could prevent a rise in the global temperature of more than 1.5° above pre-industrial levels. The completed report, Special Report on Global Warming of 1.5°C (SR15), was released on October 8, 2018 [6]. Its full title is ‘Global Warming of 1.5°. An IPCC Special Report on the impacts of global warming of 1.5°C above pre-industrial levels and related global greenhouse gas emission pathways, in the context of strengthening the global response to the threat of climate change, sustainable development, and efforts to eradicate poverty. In response to this, the IPCC and also the UK Government have recently changed their target to zero CO_2_ emissions by 2050. At the moment, over 80% of the global power demand of 18 TW (∼14.4 TW) comes from burning fossil fuels [5] which dumps ∼40 Gt of CO_2_ into the atmosphere. To replace this enormous amount of power by renewable non-carbon ‘green energy sources’ is daunting and, at the moment, almost certainly impossible.

To make my point, consider that the solution was to use only nuclear power. An average nuclear power station generates 1500 MW day and night. To generate the 14.4 TW by 2050 there would have to be 9600 functional power stations spread worldwide. Since there are about 11 100 days from now (mid-summer 2019) to 2050, then nuclear power stations would need to be built at a rate of almost one per day. Of course, the demand for energy will likely be higher in 2050 and in addition some power stations will need to be re-commissioned. For fission reactors, there is also the problem of a finite amount of nuclear fuel. The fuel problem would be overcome if the power source is nuclear fusion but at the moment there is no workable system and may never be because of technical difficulties. Even then, a nuclear fusion reactor would only produce about the same power as a fission reactor, that is 1500 MW, and they also would have to be built at a rate of one a day. I hope this simple calculation brings home the magnitude of the challenge which seems to be ignored or passed over by policy makers and their advisors.

Of course, there is no intention to strive for total nuclear power although from my own experience of talking to several policy makers in the UK, including a recent chief scientist, there is a belief that nuclear fusion will be a major player by the end of the century. However, the most recent recommendation of zero CO_2_ emissions by 2050 has been based on limiting the overall global temperature to 1.5°C, and this may be too stringent as man-made climate change skeptics would probably argue. Despite this, there is absolutely no doubt that CO_2_ levels and the global temperature are rising and ultimately humans must take heed of the risks which are being taken for the future of their species. The fact is that *Homo sapiens* has well and truly entered the Anthropocene period and our future is no longer controlled by Darwinian principles, as is the case for all other species on our planet. As such, we face a dilemma: shall we take the risk and maintain business as usual? On one hand we could accept that there will be changes but deal with them as they occur using the technology and knowledge which will be available at the time; or should we try to avoid any major changes triggered by the increasing levels of greenhouse gases, particularly CO_2_ in the atmosphere? Serious sea level increases are predicted from climate change models which in themselves are far from perfect given the complexity of global weather patterns. I, therefore, understand the difficulty that policy makers have with responding to this potential threat which would require rapid and enormous changes to maintain social order and the avoid the devastating consequences.

I think most agree that the renewable green energy source available to us of sufficient magnitude is supplied by the sun which over the year provides the Earth an average of ∼120 000 TW of power [7]. Until recently, it has been common to state that ‘*one hour of sunlight falling on our planet provides energy to power the biological and technological requirements of all humankind for one year’* [8]*.* This encouraging statement now needs to be modified to more like 90 min to take into account the increase in global power consumption over the past 13 years which is reflected in the increase in atmospheric CO_2_ levels. Furthermore, there is no doubt that significant climate changes are also occurring with record temperature levels and other climate parameters being commonplace. Surely the human race is not going to sit aside and let this continue? This is why I have turned my attention from the study of natural to artificial photosynthesis.

Until industrialisation and the closely coupled rise in human population, the uptake and storage of CO_2_ was roughly balanced by global photosynthesis (CO_2_ uptake) and respiration/fermentation (CO_2_ release). To be absolutely correct, this cycle was not perfectly balanced during most of the evolution of life on our planet. For billions of years the uptake of CO_2_ by photosynthesis exceeded that of CO_2_ release from respiration/fermentation. This extra ‘stored’ CO_2_ in the form of biomass was gradually converted into fossil fuels: oil and gas from microorganisms, while coal is derived from plant material. Today the amount stored through the eons of time is colossal. This is not appreciated by many geologists. Let me explain. The overall chemical equation for oxygenic photosynthesis is:

1 $$2{\text{H}}_{2}\text{O + C}{\text{O}}_{2}\overset{h\nu }{⟶}{\text{O}}_{2}+(\text{C}{\text{H}}_{2}\text{O})$$

This stoichiometric balanced equation shows that for every molecule of O_2_ produced, one molecule of CO_2_ is chemically reduced, and stored in organic molecules such as carbohydrate (CH_2_O). Since today there is 21% (v/v) O_2_ in the atmosphere and, as far as we know, it is derived entirely from photosynthesis, then the amount of reduced carbon on our planet is enormous. This means that there is essentially an almost unlimited supply of fossil fuels (organic molecules) although, presumably, a great deal of this reduced carbon will not be readily obtainable. (I estimate it to be in the region of 7.5 × 10^6 ^Gt). If it were all burnt (oxidised), then our planet would return to its original anaerobic state with a very high level of CO_2_ in the atmosphere and elevated surface temperatures due to the greenhouse effect. This situation would be somewhat like planet Venus today which is totally uninhabitable.

## Cellular bioenergetics

In the case of cellular bioenergetics, the initial energy converting process is the capture of solar energy by light-harvesting pigment–protein complexes embedded in the thylakoid membranes of oxygenic photosynthetic organisms (cyanobacteria, algae and various types of plants). This energy is rapidly transferred to a reaction centre where it is initially stored by charge separation across the membrane. The next stage of energy capture is using the redox properties of the charge separation state to drive the splitting of water into its elemental constituents (equation 2). In this way, molecular oxygen is released to maintain our aerobic atmosphere while the hydrogen (electrons and protons) are used as the reducing power to convert carbon dioxide into the organic molecules that constitute global biomass and the fossil fuels.

2 $$2{\text{H}}_{2}\text{O}\overset{4h\nu }{⟶}{\text{O}}_{2}+\text{4e }+4{\text{H}}^{+}$$

**Figure 1.**
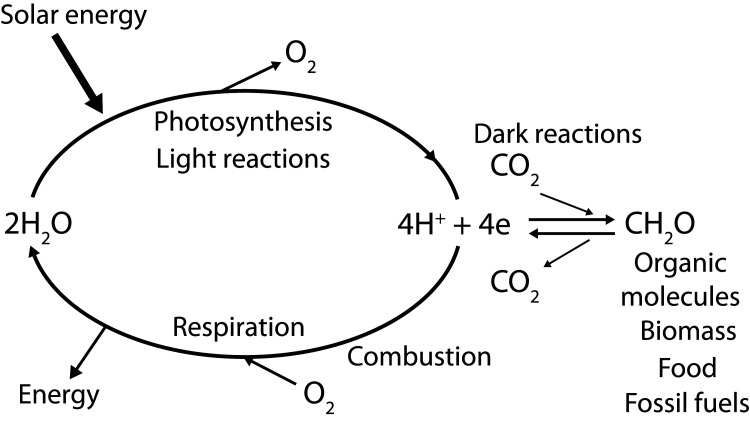
The processes of photosynthesis and respiration. The light reactions of photosynthesis (light absorption, charge separation, water splitting and electron/proton transfer) provide the reducing equivalents or ‘hydrogen’ electrons (e) and protons (H^+^) to convert carbon dioxide (CO_2_) to sugars and other organic molecules that make up living organisms (biomass), including those that provide humankind with food. The same photosynthetic reactions gave rise to the fossil fuels formed millions of years ago. The burning of these organic molecules by either respiration (controlled oxidation within our bodies) or combustion of fossil fuels is the reverse to photosynthesis, releasing CO_2_ and combining the ‘hydrogen’ back with oxygen to form water. In so doing, energy is released. Energy which originated from sunlight. Reproduced from Ref. [39] with permission from The Royal Society of Chemistry.

The water-splitting enzyme of photosynthesis is called PSII. The importance of this amazing reaction, which is the entry point for chemical energy into our planet (see Figure 1), cannot be over stated since splitting water into its elemental constituents is thermodynamically and chemically demanding, especially when achieved in a delicate biological environment and powered by the relatively low energy content of four photons of long wavelength visible light.

Thus, PSII may provide a blueprint for the development of scalable ‘artificial leaf’ technology using solar energy to extract hydrogen from water and provide a clean and renewable fuel.

**Figure 2.**
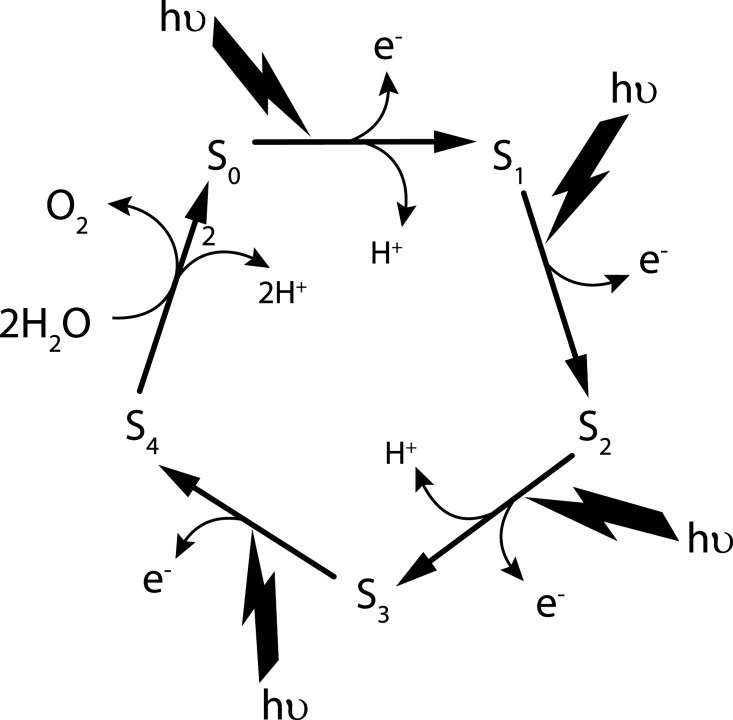
Scheme for water oxidation by PSII. The S-state cycle showing how the absorption of four photons of light (hv) drives the splitting of two water molecules and the formation of O_2_ through a consecutive series of five intermediates (S_0_, S_1_, S_2_, S_3_ and S_4_.). Reproduced with permission from [9].

The water-splitting reaction of PSII consists of five intermediate states (S_0_–S_4_), whereby four oxidising equivalents of about the same potential are sequentially accumulated at the catalytic site with the absorption of each photon (see Figure 2). The S_4_ state stores the four oxidising equivalents needed to oxidise two water molecules and in so doing reverts back to the S_0_. The oxidising equivalents are stored by four manganese (Mn) ions in the catalytic centre leading to four Mn (IV) ions in the S_3_-state just before the last photochemical step to S_4_. The precise details of this final oxidation state are currently unknown because O–O bond formation is very fast, although time-resolved studies might provide the answer. The cycle is sequentially powered by the oxidation of a reaction centre chlorophyll known as P680 generated by light-driven primary charge separation in the reaction centre of PSII coupled to a redox-active tyrosine (Y_Z_), serving as an intermediate electron carrier between P680^+^ and the Mn-cluster [9].

**Figure 3.**
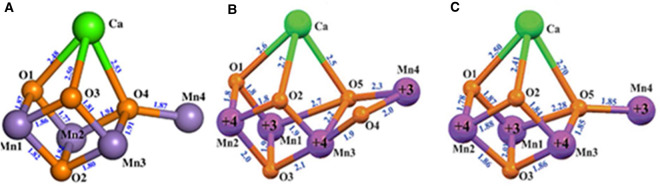
Cubane structures of the Mn_4_Ca-cluster of the water-splitting catalytic site of PSII. (**A**) The first model derived from X-ray crystallography at 3.5 Å in 2004 [10]; (**B**) improved model, derived from X-ray crystallography at 1.9 Å in 2011 [11]; (**C**) synthesised compound [12]. Adapted from Figure 6 in reference with permission [9].

In early 2004, I together with my colleagues at Imperial College London, concluded from X-ray diffraction analyses of PSII crystals at 3.5 Å resolution, that the oxygen-generating catalytic centre of PSII consisted of a Mn_3_Ca^2+^O_4_ cubane, with a fourth ‘dangler’ Mn attached to the cubane via one of its bridging oxo bonds as shown in Figure 3A [10]. Further refinement of this Mn_4_Ca^2+^O_4_ model, published 7 years later at a resolution of 1.9 Å by a Japanese group [11], confirmed this unique geometry but added one additional bridging oxo between the external dangler Mn4 and the cubane to make a Mn_4_Ca^2+^O_5_ cluster (see Figure 3B). More recently, the Mn_4_Ca^2+^O_4_ cluster has been synthesised in the absence of protein and its structure is essentially identical with that proposed from my laboratory 11 years earlier [12](compare Figures 3A,C).

**Figure 4.**
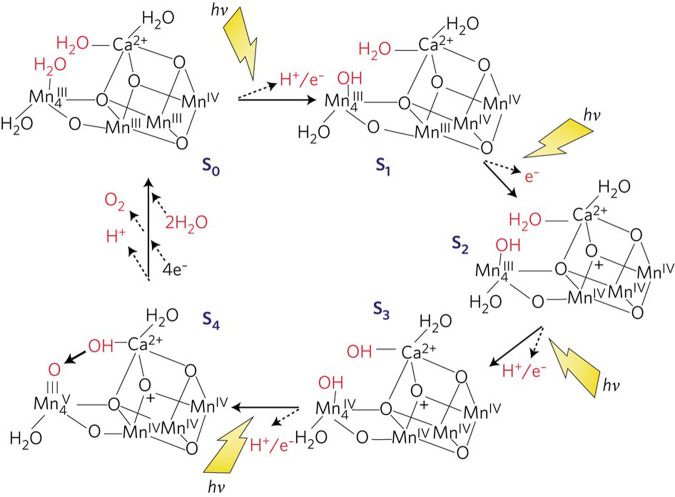
A diagrammatic representation of a mechanistic scheme for water splitting and dioxygen formation in PSII proposed by the author [14]. The substrate water molecules and products of the oxidation reactions for each S-state are shown in red. Intermediates that may exist between the S-state transitions are not depicted, nor is the possible peroxide intermediate just prior to O–O bond formation. Although the electrophilic oxo in the S_4_ state is shown attached to Mn(V) it could equally be a terminal Mn(IV)-oxyl radical species. Reproduced from [14] with permission.

Over the years there have been many postulates of the mechanism for O–O bond formation in PSII [13]. Here, and elsewhere [14], I emphasise a chemical mechanism which is consistent with the structure of the catalytic centre of PSII [10,11]. The postulate is that dioxygen formation involves a substrate water, associated with the dangler Mn4, which is deprotonated during the S-state cycle and converted to a highly electrophilic oxo. This mechanism is dependent on Mn4 being converted to a high oxidation state (possibly Mn(V) or a very reactive Mn(IV)-oxyl radical) during progression to the S_4_ state just prior to O–O bond formation. The other three Mn ions are also in high valency states Mn(IV), Mn(IV), Mn(IV) at this stage and act as a further ‘oxidising battery’ for the Mn(V)-oxo or Mn(IV)-oxyl radical species on Mn4. In this way, the reactive oxo linked to Mn4 is electron deficient, so much so that it makes an ideal target for a nucleophilic attack by the oxygen of the second substrate water bound within the coordination sphere of the Ca^2+^ (see Figure 4). The deprotonation of the substrate waters would be aided by nearby basic amino acids and by the weak Lewis acidity of Ca^2+^ and there is an extensive H-bonding network leading from the OEC to the lumenal side of complex [11,15]. A very important aspect of this mechanism is that there is proton-coupled electron transport (PCET) to avoid the build up a significant columbic charge during the cycle, although one positive charge is accumulated during the S_1_ to S_2_ transition which adds to the overall oxidising potential of the Mn_3_Ca^2+^ cubane in the S_4_ state. To experimentally prove this mechanism or any other alternative mechanism will be very difficult. This is because the substrate for this enzyme is water and the reactants are H_2_O, OH and O, which will require structural information able to detect individual protons so as to distinguish between them. Some very impressive X-ray free electron laser (XFEL) diffraction studies have recently been reported but unfortunately with resolutions far from that required to specifically reveal the details of the electron/proton reactions which underlie the water splitting and oxygen formation reactions. Nevertheless, recent XFEL measurements do provide evidence for an alternative mechanism which involves the opening of the cubane during the S_1_ to S_2_ transition which is followed by an oxyl-oxo coupling mechanism to form the O–O bond [16].

The hydroxyl/water nucleophilic attack concept for O–O bond formation (see Figure 4) which I first advocated in 2004 [10] has been demonstrated in Mn complexes synthesised to check this postulate and the possible involvement of Mn(V) [17]. This proposal also fits well with earlier suggestions [18,19]. Moreover, the nucleophilic attack mechanism can be a dominant reaction for water splitting and oxygen formation by mononuclear Ru complexes oxidised by Ce^4+^ where the electrophile is Ru(V)=O and nucleophile is solvent water [20]. Some of these Ru complexes have high turnover rates but do not match those achieved by direct oxo-radical coupling occurring in binuclear Ru complexes [21].

An interesting comparison between the catalytic mechanisms of PSII and carbon monoxide dehydrogenase, which both extract ‘hydrogen’ from water and have very similar geometries of their catalytic centres, also supports the nucleophilic mechanism on the surface of their Mn_3_Ca^2+^ and Fe_3_Ni^2+^ cubanes, respectively [22].

## Artificial photosynthesis

**Figure 5.**
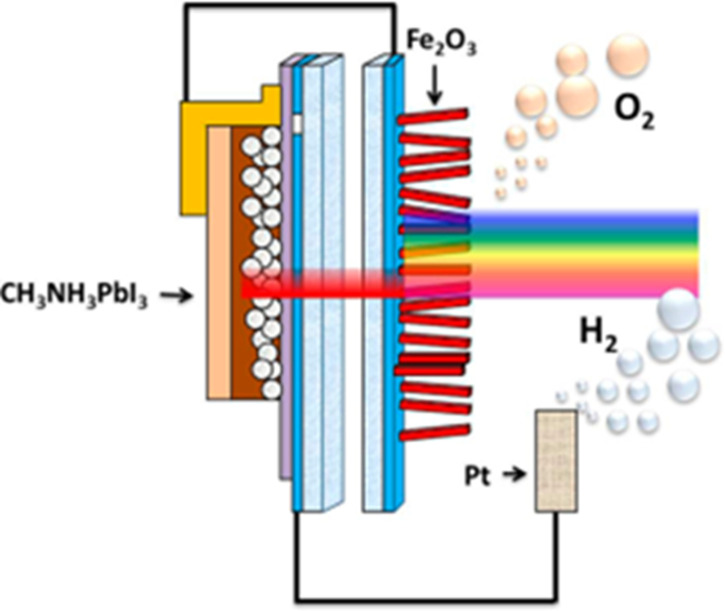
An example of an ‘artificial leaf’ device for water splitting. Diagrammatic representation of a tandem photoelectrochemical cell incorporating a hybrid organic–inorganic lead halide perovskite (CH_3_NH_3_PbI_3_) and Mn-doped haematite (Fe_2_O_3_) nanorods coated with Co-Pi and requiring no external electrical potential bias for photo-driven water splitting. Reprinted with permission from [24]. Copyright 2015 American Chemical Society.

The model of the organo-metallic oxygen-evolving cluster of PSII provides hints on the mechanism of O–O bond formation in photosynthesis, but is unlikely to be incorporated into ‘artificial leaf’ technology because of its fragility within the protein cage of the enzyme. What is required is a robust, non-toxic and abundant water-splitting catalyst. The simplest design would be to use a semiconductor to absorb visible light and generate charge separation. Ideally, the excited electrons in the conduction band should be sufficiently energetic to drive proton reduction to hydrogen while the holes left in the valence band will have a large enough oxidising potential to split water. In some cases, the water-splitting process may occur at a semiconductor surface but, since the reaction is multielectron, a bound catalyst will be required to achieve maximum rates. This co-catalyst has been employed with several semiconducting light-harvesting systems, for example, triple junction amorphous Si and hematite (Fe_2_O_3_). Hematite is particularly attractive because it is cheap, non-toxic and a good absorber of the solar spectrum. Its band gap is 2.1 eV and the light-generated holes have sufficient oxidising power (+2.0 V) to split water. Hematite requires nanostructuring to reduce the extent of recombination reactions and we have successfully developed the use of nanorods for this purpose [23]. Hematite nanorods, in combination with doping with Mn or Sn and surface passivation, are proving to help maximise these advantages. One artificial leaf device using Mn-doped hematite nanorods [24] and a Co-phosphate co-catalyst is shown in Figure 5. As the conduction band potential of hematite is not low enough to produce hydrogen without some support of an electrical bias, in this work the extra bias for hydrogen production is generated by the photovoltaic activity of a hybrid organic–inorganic lead halide perovskite tightly coupled to the photoanode. It should be noted that in photosynthesis, PSII by itself also cannot drive hydrogen production and the bias is satisfied by a second light reaction taking place in photosystem I (PSI). Another way to drive the hydrogen production in artificial photosynthesis is achieved by using semiconductors with an appropriate conduction band potential. One recent example is using earth abundant semiconductors Cu_2_ZnSnS_4_ (CZTS) as a photocathode to generate hydrogen [25].

The next step will be to incorporate a non-platinum catalyst into this ‘artificial leaf’ device which will use the protons and high energy electrons derived from the water-splitting reaction to produce hydrogen gas. Indeed, considerable progress is being made by mimicking the natural hydrogenase enzymes found in a wide variety of microorganisms [26]. Also, many metals and inorganic catalysts have been identified with activities which are almost as good as platinum as shown in volcano plots [27]. A very good class of catalysts is based on sulphides of Mo and W which can match the effectiveness of pure metal cathodes [27-29].

One successful example of the coupling of catalysts with a semiconductor for light capture and charge separation was reported by Nocera and colleagues [30]. They used a triple junction amorphous Si wafer as the semiconductor, a cobalt phosphate (CoPi) catalyst for water splitting and a NiMoZn alloy for the cathodic hydrogen-producing catalyst.

Interestingly, Cobo et al. [31] found that a metallic cobalt coated with a cobalt-oxo/hydroxo-phosphate layer in contact with the electrolyte mediated H_2_ evolution from the neutral aqueous buffer at modest over-potentials. Remarkably, it can be converted on anodic equilibration to CoPi, the O_2_ evolution catalyst. The switch between the two catalytic forms is fully reversible and corresponds to a local inter-conversion between two morphologies and compositions at the surface of the electrode. After deposition, the noble-metal free coating thus functions as a robust, bifunctional and switchable catalyst.

These and other examples of hydrogen-producing photoelectrochemical (PEC) devices are only explored at the laboratory level despite a worldwide explosion of research in this area. However, there are some interesting recent developments using particulate semiconductors for both oxygen production and hydrogen generation which are electrically linked to produce a two photon per electron tandem system [32,33]. These powder-based systems lend themselves to making functional sheets up to 1 m square and can generate hydrogen when illuminated with natural sunlight but at a low efficiency of 0.4% [34]. Another approach adopted by Li et al. [35] used an n-type organic dye to activate a Ru-based catalyst for O_2_ production, linked to a dye-activated organo-Co catalyst to generate H_2_. Here, the incident photon to current efficiency (IPCE) was as high as 25% when exposed to 380 nm light at neutral pH with no applied electrical bias.

A record efficiency of 30% for solar water splitting has been reported by Jia et al. [36] when they coupled two electrolyte polymer membrane electolysers in series with a InGaP/GaAs/GaInNAsSb triple junction solar cell. Although this is an impressive demonstration of proof of concept, it is inconceivable that GaAs-based semiconductors could be used on a massive scale due to the cost and relatively low abundance of gallium.

Given that, at present, the annual average global power consumption is ∼17 TW, with the expectation of a considerable increase by 2050, ‘artificial leaf’ technology is unlikely to have significant impact on present and near future energy supplies. However, it is always difficult to predict the emergence of new technology and the present focus of attention on this area of solar energy capture and storage is vital and must continue to grow with urgency. In this respect, there are many efforts ongoing. For example, there is the Joint Centre for Artificial Photosynthesis [37] and associated programmes funded by the US Department of Energy [38]. However, there must be more effort directed at developing scalable technology which can compete in financial terms with the relatively low cost and abundance of fossil fuels.

For large-scale energy production, the employment of PV to capture solar energy efficiently seems more realistic and then to use the photocurrent at dedicated sites to drive water splitting using a new generation of efficient low-cost electrochemical cells. Currently, electrolysis of water to obtain ‘green’ hydrogen on a large scale is expensive. However, as mentioned above, low-cost and non-toxic materials are being identified as alternatives to platinum for the electrochemical splitting of water. The establishment of a new generation of electrolysers resulting from this research can also be used to store electricity derived from other sources such as wind-, hydro-, wave- and nuclear-power. But they must have very good turnover rates to cope with high levels of electricity supplies. The more tightly coupled solar-driven systems may have application at the personal level, however, such as individual houses and as a fuel source in remote areas in under-developed countries.

## Epilogue

Since starting this short paper in 2019, there has been a dramatic increase in publicity regarding the problem of climate change with several millions of people protesting about the lack of action taken by policy makers over the past 50 years (250 000 alone demonstrated in New York and heard live the speech by Greta Thunberg). On the other hand, as I have tried to emphasise in the first section of this paper, we need to provide energy and food for a rising global population and satisfy their other demands (including luxuries, often disposable and with a short life time). The problem all comes down to providing an adequate energy supply from decarbonised and renewable fuel. So, although most of the general public (and policy makers) are now more aware of man-made climate change, how to solve the problem is not talked about. The problem is creating renewable energy on an enormous scale.

## Perspective

- A sustainable energy supply is needed to reduce global CO_2_ emissions to help mitigate climate change. Hydrogen gas, generated by the electrolysis of water, is an excellent clean energy carrier with a high energy density in its compressed form and so ideal for transport systems including automobiles, buses, motor cycles, ships and aircraft.
- Using the energy of sunlight to split water into its elemental constituents is a potential route for the large-scale production of hydrogen gas. Studies on PSII have revealed the structure of the manganese/calcium inorganic catalyst that is used by nature to split water.
- Rapid progress is being made to develop synthetic systems that efficiently use solar energy to produce hydrogen and oxygen from water. Many are based on the principles used by nature to split water.

## Open Access

Open access for this article was enabled by the participation of Imperial College London in an all-inclusive Read & Publish pilot with Portland Press and the Biochemical Society under a transformative agreement with JISC.

## Abbreviations

IPCC: Intergovernmental Panel on Climate Change
PSII: photosystem II
XFEL: X-ray free electron laser
